# Differential Effects of Hypoglycemia and Excitotoxic Signals on SN56 Septal Cholinergic Neuronal Cells

**DOI:** 10.3390/cells15110960

**Published:** 2026-05-22

**Authors:** Sylwia Gul-Hinc, Andrzej Szutowicz, Anna Ronowska, Agnieszka Jankowska-Kulawy

**Affiliations:** Department of Laboratory Medicine, Medical University of Gdańsk, 80-210 Gdańsk, Poland; sylwia.gul-hinc@gumed.edu.pl (S.G.-H.); anna.ronowska@gumed.edu.pl (A.R.)

**Keywords:** hypoglycemia, cholinergic neuronal cells, acetyl-CoA, neurotoxicity, pyruvate dehydrogenase, ATP-citrate lyase, choline acetyltransferase

## Abstract

Glucose is the principal energy substrate for the brain. Hypo- and hyperglycemic episodes frequently occur in senescent people, contributing to functional and structural impairment of brain neurons and causing cognitive deficits in this population. In this study, we investigate whether long-term changes in the extracellular concentration of glucose affect viability and transmitter functions of septum-derived SN56 cholinergic neuronal cells through alterations in acetyl-CoA availability. Cells with low cholinergic expression (NCs) and cAMP/retinoic acid-induced high cholinergic expression (DCs) were investigated. Hypoglycemia brought about similar (approximately 20–30%) decreases in pyruvate dehydrogenase complex (PDHC) and ATP-citrate lyase (ACLY) activities and a 65% decline in lactate dehydrogenase (LDH) activity in NCs and DCs. Choline acetyltransferase (ChAT) and LDH activities in DCs were about 3–8 and 1.7–2.4 times higher than in NCs over the tested glucose concentration range, respectively. DCs appeared to be more resistant than NCs to hypoglycemia, as evidenced by lower glucose IC**_50_** values for cell count and intracellular LDH activity. On the other hand, some of functional properties of DCs, such as the cholinergic phenotype and their plasma membrane functions (trypan blue exclusion, TB+), were found to be more sensitive to hypoglycemia than those of NCs, as demonstrated by the higher IC**_50_** for glucose in DCs. Acetyl-CoA levels in DCs were 40% lower than in NCs, and decreased by about 25% with increasing hypoglycemia in both cell types. The cytotoxic effects of amyloid-β**_25–35_** (Aβ) and sodium nitroprusside (SNP; NO generator) were also tested. In 25 mM glucose medium, these toxic compounds exerted greater detrimental effects on DCs than on NCs. In contrast, in 1 mM glucose, more evident cytotoxicity of SNP and Aβ was observed in NCs. These data suggest that the higher rate of glycolysis in differentiated cholinergic septal neurons may be a protective mechanism against hypoglycemia.

## 1. Introduction

The adult brain utilizes about 150 g glucose per day as its almost exclusive energy substrate. This corresponds to 20% of the whole-body glucose uptake under resting conditions. Accordingly, the average rate of energy metabolism in the brain is 10 times faster than that of other organs. Neuronal demands for glucose and oxygen are so high due to neurons’ functional action potentials with a frequency of 10–50 Hz. Therefore, severe/acute deficits in glucose/oxygen supplies trigger instant symptoms of sleepiness, vertigo, loss of awareness, coma, excitotoxic reactions such as trembling or epilepsy, and delayed inflammatory reactions [1,2,3]. These deficits, depending on the magnitude and localization, may cause cell death and/or dysfunction in different neuronal groups [4,5,6,7].

Cholinergic neurons in the human central nervous system play a key role in cognitive and motor functions. They also exert neuromodulatory effects on other brain neurotransmitter systems. Cholinergic transmission is pivotal in the analysis and integration of visual and auditory sensory signals, which are converted into conscious behaviors, conditional reactions, cognition, and memory formation processes [8]. Cholinergic neurons are more susceptible than other brain neuronal cells and glial cells to several pathological inputs, which are responsible for the onset and development of several neurodegenerative diseases including cholinergic encephalopathies such as Alzheimer’s (AD), Parkinson’s disease, and Wernicke–Korsakoff syndrome [9,10]. Moreover, brain cholinergic neurons with different phenotypes may display differential susceptibility to similar pathological inputs [9,11]. Lesions in basal cholinergic neurons that innervate the hippocampus and several regions of the cerebral cortex may cause diverse cognitive deficits [12]. Studies on cholinergic neuronal cells cultured in standard, high-[glucose] DMEM have demonstrated that specific phenotypes are more prone to neurodegenerative insults [11,13].

Disturbances to energy metabolism in human cholinergic encephalopathies are also well documented. Impaired glucose metabolism associated with altered activity of oxidative enzymes such as pyruvate and oxoglutarate dehydrogenases and aconitase are typical hallmarks of Alzheimer’s disease (AD) [14,15]. This may be because cholinergic neurons utilize glucose-derived acetyl-CoA not only for energy production and lipid synthesis, but also for ACh synthesis. Thus, the high vulnerability of cholinergic neurons to neurodegenerative inputs may be associated with shortages in their acetyl-CoA supply [16,17].

Clinical reports demonstrate that transient hypoglycemic or/and hypoxic episodes frequently occur in both neonatal and elderly populations [18,19]. In this study, mouse septum-derived neuroblastoma SN56 cells were employed as an in vitro model for studying the effect of hypoglycemic insult on cholinergic neurons [11,17]. Hypoglycemic episodes not only impair key energy metabolic functions in neurons, but may also trigger remote effects such as excessive Aβ accumulation or tau hyperphosphorylation [4,6,20]. In contrast, streptozotocin-induced diabetes in rats causes elevated pyruvate utilization, acetyl-CoA levels, and rates of ACh synthesis and release in isolated brain nerve terminals [21]. However, diabetic subjects display an increased risk for AD [22]. In agreement with this observation, in vitro excitotoxic Zn/NOO^−^ excess has been shown to adversely affect key functions of cholinergic neuronal SN56 cells, even in DMEM containing the optimal glucose concentration of 25 mM [11,23,24]. Our experiments revealed that under diverse excitotoxic conditions, the viability and transmitter function of cholinergic neuronal cells are strongly correlated with their acetyl-CoA content [25]. However, there are no data on whether such interdependencies exist under the chronic or incidental hypoglycemic conditions that occur in different pathologies. Hypoglycemia is also a common and widely investigated condition that affects the brain in pre-term and neonatal children [19]. However, neither diverse clinical studies, in vivo animal experiments, nor cell culture experiments on hypoglycemia have provided any data on acetyl-CoA levels in these conditions despite their known importance in brain pathologies [17,19,26,27].

Therefore, the aim of this work was to investigate whether and how lowering the [glucose] in culture medium affects enzymes involved in acetyl-CoA and ACh metabolism and structural integrity in cholinergic septal neurons, which are early, prevalent targets for diverse cholinergic encephalopathies [9,12]. To this end, we used media with 1–25 mM glucose to cover the whole range of extreme clinical conditions. The data provide evidence that hypoglycemia may evoke alterations in [acetyl-CoA] and LDH levels, contributing to the phenotype-dependent differential effects on the viability of brain septal cholinergic neurons.

## 2. Materials and Methods

### 2.1. Reagents

Unless otherwise specified, all biochemicals were obtained from Sigma-Aldrich (Poznań, Poland). Acetyl-CoA [1-^14^C-acetyl] (4 mCi/mmol) was purchased from Perkin-Elmer (Boston, MA, USA) and cell culture disposables were obtained from Sarsted (Stare Babice, Poland). Amyloid-β (25-35) was purchased from Bachem (Heidelberg, Germany); to obtain aggregated Aβ, this peptide was dissolved in a sterile 50 mM saline buffer solution and kept for 96 h at 37 °C.

### 2.2. Cell Culture

**Figure 1:**
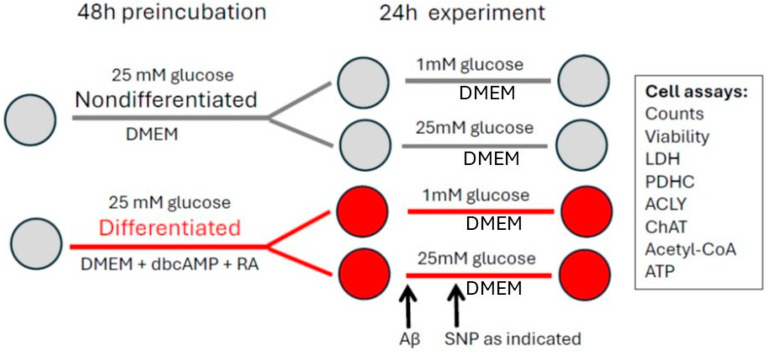
Experiment protocol for investigating effect of glycemia, Aβ, and SNP on acetyl-CoA metabolism in nondifferentiated and differentiated SNS56 murine septal cholinergic neuronal cells. Cells from passages 20–46 with stable expression of ChAT and PDHC were used.

The SN56 neuroblastoma cells used in the experiments were generated by fusing N18TG2 neuroblastoma cells with murine (strain C57BL/6) neurons derived from postnatal day 21 mouse septum (gift from Prof. J.K. Blusztajn, Boston, MA, USA) [28]. The cells were cultured in 10 cm diameter Petri dishes in Dulbecco’s Modified Eagle Medium (DMEM, Sigma-Aldrich D5671) containing 25 mM glucose, 4.0 mM L-glutamine, an antibiotic mixture (250 ng fungizone, 50 µg streptomycin, and 50 IU penicillin per 1 m), and 10% fetal bovine serum, which is the standard medium used for this cell line [26,27]. In the preliminary step, the cells were seeded at a density of 26 × 10**^3^**/cm**^2^** and cultured for 48 h in a 5% CO_2_ atmosphere at 37 °C in the absence (NCs) or presence of 1 mM dbcAMP and 0.001 mM all-trans retinoic acid (RA) as differentiation factors (DCs) until they reached subconfluency (50–70 × 10**^3^** cells/cm**^2^**). These factors increased the cholinergic properties of the neuroblastoma cells, resulting in morphologic maturation and several-fold increases in choline acetyltransferase (ChAT) activity and acetylcholine (ACh), vesicular ACh transporter, and high-affinity choline transporter levels (Figure 1) [11,13,27].

Next, the NC/DC media were removed and replaced with the experimental basal DMEM (Sigma-Aldrich D5030) supplemented with glucose at concentrations of 1–25 mM, and 120–110 mM NaCl to maintain the osmolality of all final media at 295–298 mOsm/kg H_2_O. The media also contained 2.0 mM L-glutamine, 40 mM NaHCO_3_, an antibiotic mixture (250 ng fungizone, 50 µg streptomycin, and 50 IU penicillin per 1 m), and 10% fetal bovine serum without any differentiation factors. The cells were incubated for another 24 h; neurotoxic agents (0.001 mM aggregated (aged) Aβ**_25–35_** and 0.4 mM sodium nitroprusside (SNP), a precursor of NOO^−^) were added for last 24 or 16 h, respectively (Figure 1).

The experimental media were removed, and the cells were rinsed with ice-cold 140 mM NaCl containing 5 mM KCl, 1,7 mM Na-K phosphate buffer (pH 7,4), and 5 mM glucose. The cells were harvested using 10 mL of the buffer and collected by centrifugation at 200× *g* for 8 min The supernatant was removed and the cells were suspended in 320 mM sucrose in 10 mM HEPES buffer (pH 7.4) with 0.1 mM EDTA to achieve a protein concentration of about 10 mg/mL. Immediately after collection, cell aliquots were taken to assess cell counts, viability, and metabolic parameters. To measure enzyme activities, the cell suspension aliquots were kept frozen at −20 °C for 2–7 days before the assays were performed.

### 2.3. Viability and Cell Counts

The cell suspensions were mixed with an equal volume of an isotonic 0.4% trypan blue solution. The total number of cells and fractions of viable and nonviable cells were counted after 2 min using a Fuchs–Rosenthal hemocytometer under a light microscope [29].

### 2.4. Enzyme Assays

The samples were diluted with 0.2% (*v*/*v*) Triton X-100 to achieve the appropriate protein concentration. LDH activity was assayed using a spectrophotometric method. ChAT activity was measured using a radiometric method and [1-^14^C] acetyl-CoA as the substrate [30]. PDHC activity was measured using the trapping method with citrate synthase [31].

### 2.5. Protein Assay

The samples’ protein concentrations were measured using the Bradford method (1976) [32] and human ɤ-globulin as the standard.

### 2.6. Acetyl-CoA Assay

The samples for the whole-cell acetyl-CoA assay were adjusted with 0.9% NaCl to a protein concentration of 100–150 µg per sample and then centrifuged at 200× *g* for 3 min. The supernatant was removed and the cell pellet was deproteinized by suspending it in a small volume of 5 mM HCl. The samples were kept frozen at −20 °C for 2–7 days before they were used in the assay. To assess acetyl-CoA levels, the deproteinized extracts of whole cells were treated with a maleic anhydride solution in ethyl ether for 2 h to remove CoA-SH. The cycling reaction was carried out for 100 min at 30 °C in 0.1 mL of medium containing 1.9 mM acetyl phosphate, 1.2 mM oxaloacetate, 0.72 IU phosphotransacetylase, and 0.12 IU citrate synthase. The cycling reaction was stopped by heating the sample to 100 °C for 10 min, and the formed citrate was measured [33].

### 2.7. Statistics

The results are shown as the mean ± SEM from 3–10 independent culture experiments. Calculations of glucose concentration–response relationships ([IC**_50_**/AC_50_]) were performed using non-linear regression analysis in Graph Pad Prism Software 5.0 (Graph Pad Software Inc., La Jolla, CA, USA) by fitting the data to log[glucose] data. Differences between glycemia-derived data were estimated using one-way ANOVA followed by a post hoc Dunnett’s test. Differences between corresponding NC and DC data were assessed using Student’s *t* test. Correlations between different variables were determined using linear regression with regression coefficients used as indicators of statistical significance.

## 3. Results

### 3.1. Effect of Hypoglycemia on SN56 Cells

**Figure 2:**
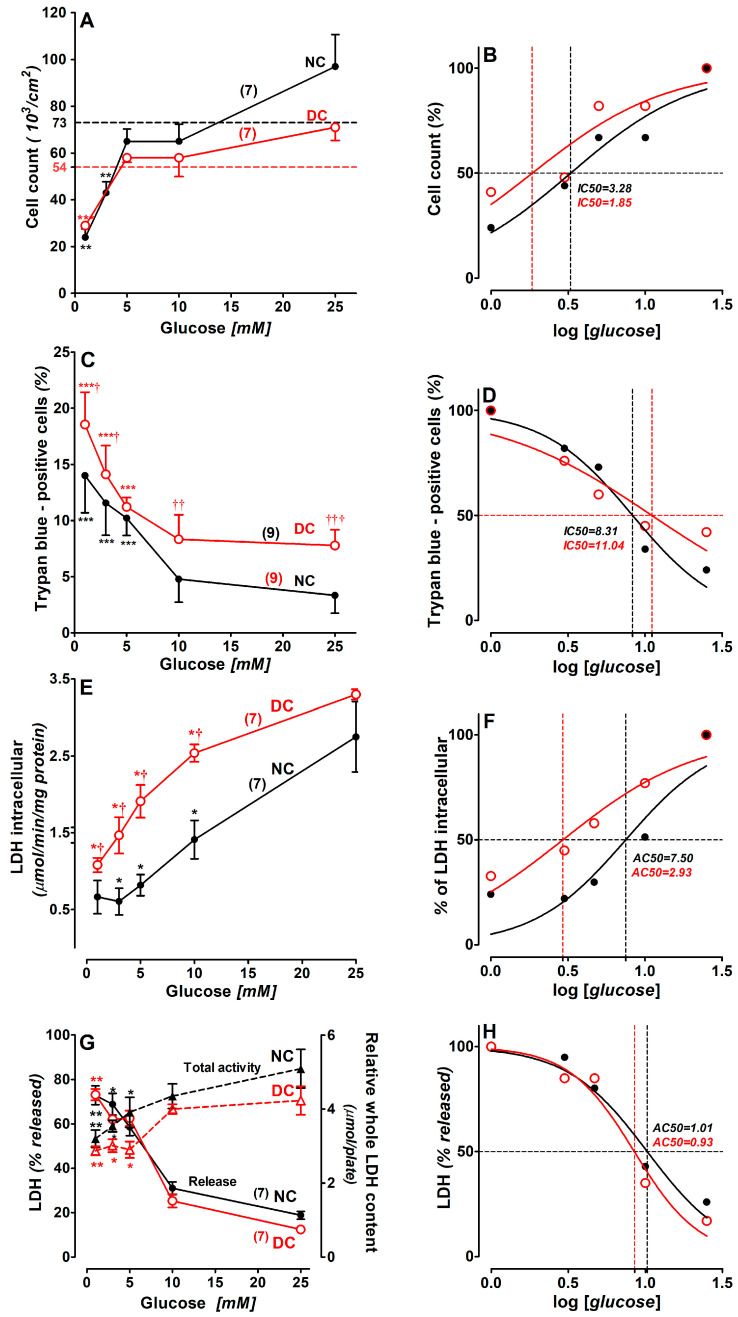

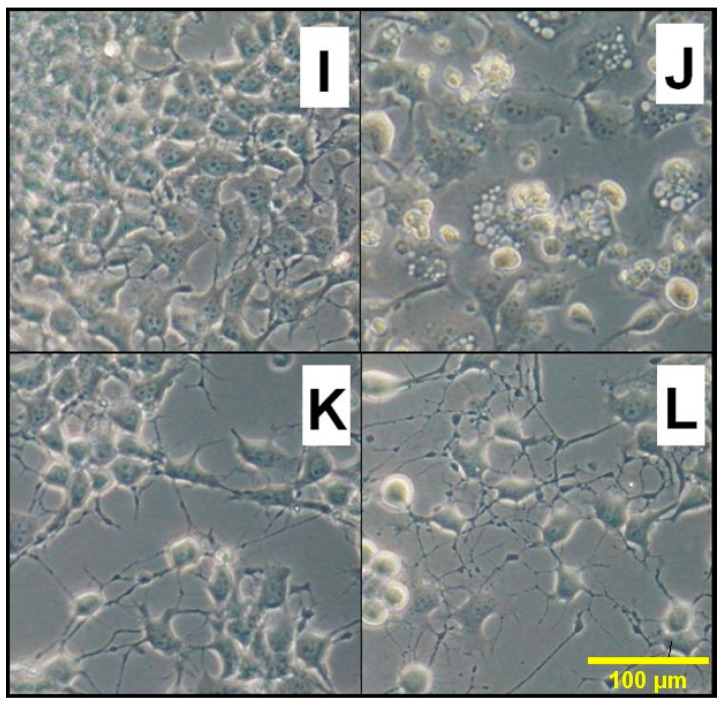
Effects of glycemia on SN56 cells. Concentration-dependent effects of glycemia on cholinergic SN56 NCs ● and DCs **○:** (**A**) plots of 72 h (solid lines) and 48 h (dotted horizontal lines) culture cell counts; (**B**) semi-log response plots of cell count vs. [glucose]; (**C**) viability (trypan blue exclusion test); (**D**) semi-log response of TB positivity vs. [glucose]; (**E**) intracellular LDH activity vs. [glucose]; (**F**) semi-log response plots of intracellular LDH activity vs. [glucose], (**G**) fractional LDH release to the medium and medium LDH activity (dashed lines) vs. [glucose]; (**H**) semi-log response plots of released LDH vs. [glucose]. Data are the mean ± SEM from 7–10 independent experiments (indicated by the numbers in brackets). Compared to respective 25 mM glucose control: * *p* < 0.05, ** *p* < 0.01, *** *p* < 0.001; compared to respective NCs: **^†^** *p* < 0.05, **^††^** *p* < 0.01, **^†††^** *p* < 0.001. Morphology of SN56 neuronal cells grown in DMEM: (**I**) NCs in 25 mM glucose; (**J**) NCs in 1 mM glucose (**K**); DCs in 25 mM glucose; (**L**) DCs in 1 mM glucose. Photographs are representative of 5 experiments; magnification: 400×.

The glycemia in extracellular body compartments in vivo may vary widely under different physiological and pathological conditions. In particular, acute hypoglycemia may exert rapid and often fatal effects on neurons as they require about 10 times more glucose under resting conditions compared to other tissues [3,7]. During the preliminary 48 h of culture in standard DMEM, the density of SN56 NCs and DCs increased to about 73 × 10^3^/cm^2^ and 54 × 10^3^/cm^2^, respectively (Figure 2A). After 72 h of culture in standard 25 mM glucose medium, the number of NCs and DCs increased to 97 × 10^3^ and 71 × 10^3^/cm^2^, corresponding to 4- and 3-fold increases, respectively (Figure 2A). The decrease in [glucose] to 5 mM did not cause any significant alterations in cell count or proliferation rate. Reduction of glycemia to 1 mM resulted in 75 and 59% lower NC and DC counts (Figure 2A). The half-maximal activation concentrations for glucose (AC**_50_**) for NCs and DCs were 3.28 and 1.85 mM, respectively (Figure 2B). Under the same conditions, the fractions of nonviable, trypan-blue-retaining (TB+) NCs and DCs increased from 3 to 14% and from 7 to 18%, respectively (Figure 2C). The glucose IC**_50_** values for TB retention in NCs and DCs were 8.31 and 11.04 mM, respectively (Figure 2D).

Relative LDH release from cells into the medium is an indicator of their impairment/death. On the other hand, intracellular LDH is a marker of plasma membrane integrity and glycolytic activity of living cells [34,35]. Hypoglycemia (1 mM) resulted in similar (approximately 65%) decreases in LDH activity in both groups. However, at all glycemia levels, the specific activity of intracellular LDH in DCs was 70–140% higher than that in NCs (Figure 2E). The glucose IC**_50_** for decreasing intracellular LDH activity in DCs was over two times lower than that in NCs (Figure 2F). On the other hand, 1 mM glucose (hypoglycemia) increased the amount of extracellular LDH from 12% to about 72% of the whole LDH pool in both NCs and DCs (Figure 2G). The glucose IC**_50_** for LDH release from DCs was about 80% higher than that in NCs (Figure 2H). The overall LDH activity in DCs and NCs cultured in media with 1 mM glucose were about 32–38% lower than in those cultured in 25 mM glucose (Figure 2G).

In DMEM with 25 mM glucose, NCs grew densely with short and scarce protrusions (Figure 2I). Hypoglycemia caused the formation of peripheral vacuoles in the cell cytoplasm and membrane malformations (Figure 2J). The 48 h preliminary differentiation of SN56 cells with dbcAMP + RA [25] caused the formation of axons, synapse-like connections, and dendritic spines, resembling a mature brain neuronal network. Mature DCs were maintained in standard 25 mM glucose medium for the subsequent 24 h (Figure 2K). Exposure to 1 mM hypoglycemia for 24 h resulted in thinning of the protrusions but no other notable changes in morphology of the surviving DCs despite a marked increase in the death rate (Figure 2A,C,E,L).

### 3.2. Effect of Hypoglycemia on Enzymes Involved in Acetyl-CoA Metabolism in SN56 Cells

**Figure 3:**
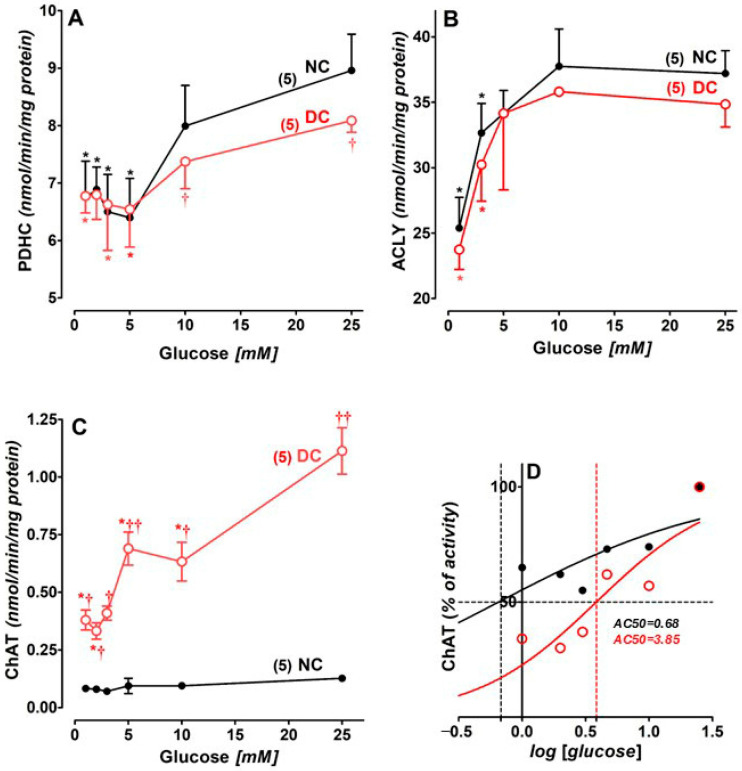
Concentration-dependent effects of glycemia on specific activities of enzymes involved in acetyl-CoA metabolism in cholinergic SN56 NCs (●) and DCs (○): (**A**) PDHC; (**B**) ACLY; and (**C**) ChAT. (**D**) Semi-log response plots of ChAT activity vs. glycemia. Data are the mean ± SEM from 5 independent experiments. Compared to respective 25 mM glucose control: * *p* < 0.05; compared to respective NCs: **^†^** *p* < 0.05; **^††^** *p* < 0.01.

PDHC is a key multi-enzyme complex that synthesizes the bulk of the acetyl-CoA in the mitochondrial compartment in neurons [17]. Hypoglycemia caused 17 and 24% decreases in PDHC activity in DCs and NCs, respectively (Figure 3A). In 25 mM glucose medium, PDHC activity in DCs was slightly lower than that in NCs, but at lower glucose concentrations, no significant differences between the groups were found (Figure 3A). ACLY is a key enzyme in the generation of acetyl-CoA in the cytoplasmic compartment [17]. No significant differences in ACLY activity between DCs and NCs were observed, though a 33% decline in its activity was observed under severe hypoglycemia conditions (Figure 3B).

Differentiation resulted in approximately three- and eight-fold increases in ChAT activity in SN56 cells grown in 1 and 25 mM glucose medium, respectively (Figure 3C). Alterations in glycemia did not affect the activity of ChAT in NCs. On the other hand, in DCs, a reduction in DMEM [glucose] from 25 to 1 mM resulted in a 66% decrease in ChAT activity from 1.11 to 0.37 nmol/min/mg protein (Figure 3C). Thus, the glycemia AC**_50_** for DC ChAT activity was about 3.85 mM compared to 0.68 mM in NCs (Figure 3D).

### 3.3. Effect of Hypoglycemia on Energy Metabolite Levels in SN56 Cells

**Figure 4:**
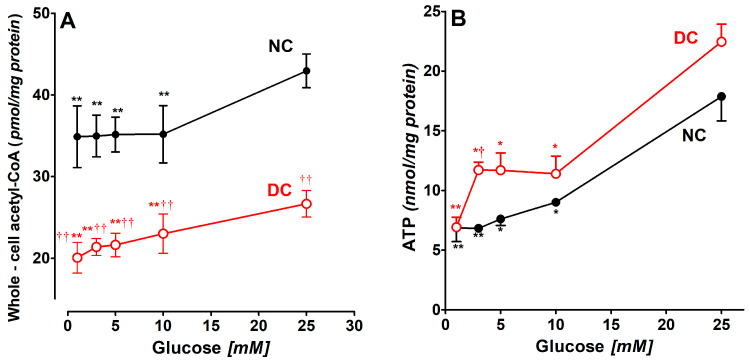
Glycemia effects on energy metabolite levels. Concentration-dependent effects of glycemia on (**A**) acetyl-CoA and (**B**) ATP levels in cholinergic SN56 NCs and DCs. Data are the mean ± SEM from 5 independent experiments. Compared to respective 25 mM glucose control: * *p* < 0.05, ** *p* < 0.01, compared to respective NCs: **^†^** *p* < 0.05; **^††^** *p* < 0.01.

The acetyl-CoA levels in DCs were about 40% lower than those in NCs, irrespective of glucose level in the growth medium (Figure 4A). Hypoglycemia resulted in 20 and 25% decreases in whole-cell acetyl-CoA levels in NCs and DCs, respectively (Figure 4A). This was accompanied by a nearly proportional decrease in viability, reflected by an increase in the TB+ cell fraction (Figure 2B,C), and a non-proportionally higher death rate, based on lower cell counts and intracellular LDH activity (Figure 2A,E).

On the other hand, a decrease in medium [glucose] from 25 to 1 mM resulted in a decrease in ATP concentrations down to 40 and 30% of the maximum detected in NCs and DCs, respectively (Figure 4B). The ATP levels in NCs were 20 and 40% lower than those in DCs when cultured in 5 and 3 mM glucose DMEM, respectively.

### 3.4. Glycemia-Dependent Effects of NO and Aβ on Viability and Parameters of Acetyl-CoA Metabolism

Extensive depolarization of glutamatergic terminals during focal or general hypoxia, hypoglycemia, or other brain pathologies induces sustained depolarization of glutamatergic neurons [6,11,24]. Excessive amounts of glutamate and Zn are released from glutamatergic terminals, triggering NMDA and other activating receptors, resulting in Ca/Zn overload in postsynaptic neurons [17]. This induces extensive activation of NO synthases and excessive accumulation of NO and nitrogen and oxygen free radicals, which impair postsynaptic neurons, including cholinergic ones. If such disturbances are chronic, they activate the Aβ-generating proteolytic pathway for APP [36,37]. In addition, our past data revealed that DCs grown in standard 25 mM glucose DMEM are more susceptible to diverse cytotoxic signals than NCs [19,38]. Therefore, we studied the effects of Aβ and the NO donor SNP on DCs as putative early and late neurotoxic signals that might accompany hypoglycemic conditions, respectively [2,4,25].

In DMEM with 25 mM (standard) and 1 mM (hypoglycemic) glucose, 0.4 mM SNP reduced NC and DC counts by about 55 and 45%, respectively (Table 1, section A). On the other hand, 0.001 mM Aβ**_25–35_** (Aβ) did not have any effects, both when applied alone or in combination with SNP (Table 1, section A).

In 25 mM glucose DMEM, SNP alone increased the fraction of TB+ NCs to 37% and that of TB+ DCs to 77% (Table 1, section B). In contrast, in 1 mM glucose, SNP increased the TB+ fraction to 97% in NCs and to 35% in DCs. Aβ alone did not significantly affect viability under any conditions (Table 1, section B). However, in 1 mM glucose DMEM and in the presence of SNP, Aβ increased the TB+ fraction of NCs and DCs to 100 and 57%, respectively (Table 1, section B). In contrast, in 25 mM glucose under cytotoxic conditions, the number of viable DCs was lower than that of NCs (Table 1, section C).

In standard 25 mM glucose medium, SNP treatment resulted in 28% and 24% inhibition of PDHC activity in NCs and DCs, respectively. On the other hand, in 1 mM glucose medium, SNP induced 50 and 35% inhibition of this enzyme in NCs and DCs, respectively (Table 1, section D). Aβ alone did not affect PDHC activity under any experimental conditions. Only in 25 mM glucose medium did Aβ aggravate SNP inhibition of PDHC activity in NCs to 69% (Table 1, section D).

In NCs cultured in 25 mM glucose, Aβ, SNP, and SNP+Aβ decreased the whole-cell [acetyl-CoA] by 35, 60, and 50%, respectively (Table 1, section E). No statistically significant effects were observed in NCs cultivated in 1 mM glucose medium. On the other hand, in DCs grown in 25 mM glucose, Aβ and SNP decreased the acetyl-CoA level by 22 and 62%, whereas SNP+Aβ increased it to 77%. In DCs incubated in 1 mM glucose, SNP and SNP+Aβ exerted weaker, non-additive 51–55% suppression (Table 1 section E). Irrespective of condition, all acetyl-CoA levels in DCs were significantly lower than those in the corresponding NCs (Figure 4A; Table 1, section E).

Exposure of SN56 NCs to SNP and SNP + Aβ in 25 mM glucose medium caused 40 and 70% suppression of the already low basal activity of ChAT (Table 1, section F). No significant alterations in NC ChAT activity was observed in 1 mM glucose medium. In DCs cultured with 25 mM glucose, SNP inhibited the already high basal activity of ChAT by 35%, whereas Aβ did not have an effect (Table 1, section F)). In 1 mM glucose medium, SNP alone resulted in 42% suppression of ChAT activity in DCs. Neither Aβ alone nor Aβ with SNP altered ChAT activity in NCs (Table 1, section F).

## 4. Discussion

Since the early 1960s, neuronal cell cultures have been mainly performed in DMEM containing the standard 25 mM glucose concentration, which is optimal for cell growth in vitro; however, this concentration corresponds to extreme diabetic conditions in vivo [5,11,24,39,40,41]. Our experiments revealed that under these conditions, cholinergic SN56 DCs were more susceptible to neurotoxic inputs than NCs due to their high demand for acetyl-CoA for ACh synthesis [17,25]. Therefore, we used a wide range of glucose concentrations down to 1 mM, which reflects the diverse extreme hypoglycemic states seen in clinical practice [39,40]. For instance, transient hypoglycemic episodes in the course of diabetes treatment, central or focal hypoxia, brain artery obstruction, or liver failure are common in aging populations, and may significantly limit the input of glucose and oxygen into the brain [41,42]. In addition, in pre-term infants and newborns, hypoglycemia accompanies several inherited and acquired metabolic conditions [43,44]. It should be noted that a blood glucose level <1.8 mM in these conditions corresponds to a <1.2 mM concentration in the cerebrospinal fluid [44]. Hypoglycemia disrupts brain ontogenesis, often resulting in different cholinergic encephalopathies [45]. Hence, the 1 mM glucose concentration tested here is relevant to specific clinical conditions.

**Figure 5:**
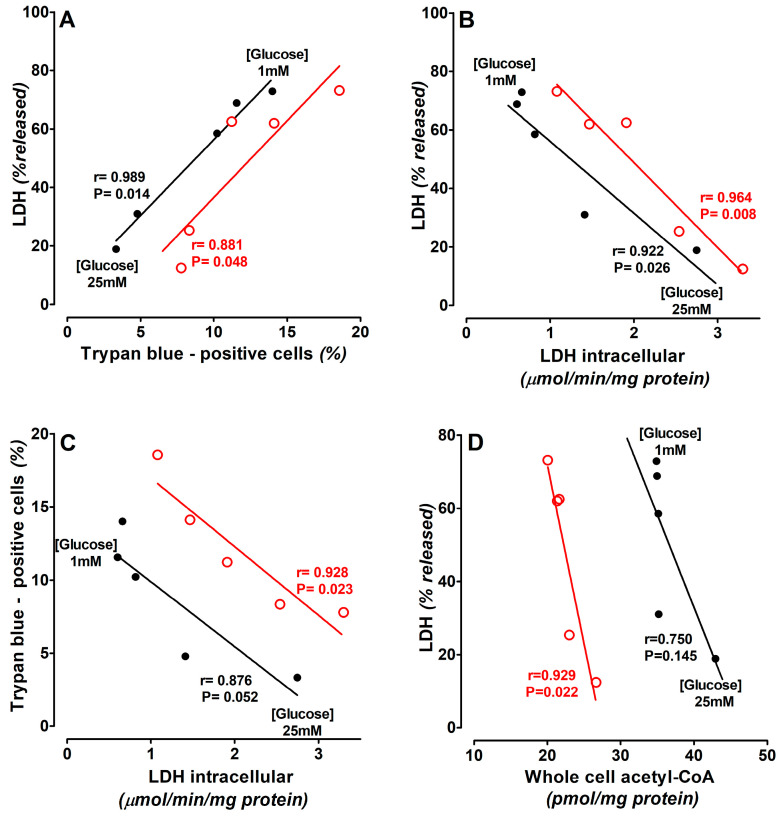
Correlation plots between SN56 cell injury parameters and acetyl-CoA level vs. glycemia (25–1 mM). (**A**) Extracellular LDH vs. TB+ cell fraction; (**B**) extracellular LDH vs. intracellular LDH; (**C**) TB+ fraction vs. intracellular LDH; (**D**) extracellular LDH vs. whole-cell acetyl-CoA. Data for calculations taken from Figure 2B,C, Figure 3D and Figure 4A.

**Figure 6:**
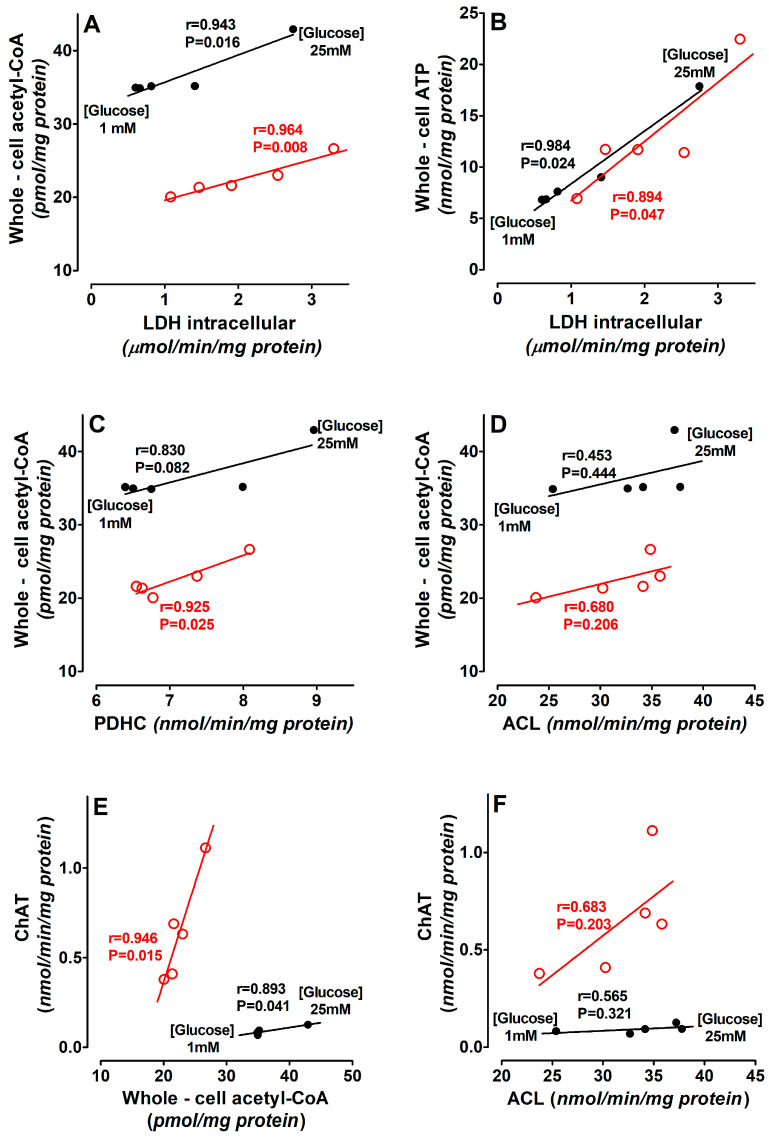
Correlation plots between acetyl-CoA and ATP levels vs. enzyme activities, which are linked with their synthesis and utilization rates, in NC and DC SN56 cells grown in media with glucose concentrations of 25–1 mM. (**A**) Whole-cell acetyl-CoA vs. intracellular LDH; (**B**) whole-cell ATP vs. intracellular LDH; (**C**) whole-cell acetyl-CoA vs. PDHC activity; (**D**) whole-cell acetyl-CoA vs. ACLY activity; (**E**) ChAT activity vs. whole-cell acetyl-CoA; (**F**) ChAT activity vs. ACLY activity. Data taken from Figure 2, Figure 3 and Figure 4.

In contrast, hyperglycemic and diabetes-related conditions may stimulate acetyl-CoA and ACh metabolism in brain neurons in vivo [20,41]. However, the putative causal links between cholinergic hyperactivation and the onset of diabetic encephalopathy have not been tested. On the other hand, hypoglycemia may generate faster and more severe neurodegenerative effects in vivo due to the decreased glycolysis rate resulting in limited provision of pyruvate substrates for PDHC [46]. These effects are also accompanied by excitotoxicity due to excessive depolarization of glutamatergic neurons [47], which results in acute ATP deficits and the loss of postsynaptic neuron membrane action potentials (Figure 3A,B and Figure 4) [3,39]. Our findings are in agreement with past in vivo and in vitro data demonstrating that chronic moderate hypoglycemia suppresses neuronal cell growth, reduces viability, and increases cell death (Figure 2 and Figure 3) [48,49,50]. The separate but parallel reciprocal correlation plots of LDH activity and TB+ vs. metabolic parameters in NCs and DCs prove that cAMP/RA-evoked activation of ACh transmitter metabolism generated neuronal populations that differ in quantitative but not qualitative responses to hypoglycemia (Figure 5 and Figure 6).

The existence of putative causative links between metabolic/enzymatic and viability parameters is supported by their significant direct and reciprocal correlations (Figure 5 and Figure 6).

However, the light microscopy images demonstrated that hypoglycemia, despite inducing the loss of SN56 DCs, did not abolish the dbcAMP/RA-pre-induced mature morphologic phenotype of the surviving neurons (Figure 2I–L). They still showed the ramified dendritic/axonal morphology and network of interconnections that formed during the preliminary step (Figure 2K,L). This could be explained by the fact that neuronal maturation is an irreversible process; therefore, adverse conditions induce degeneration and disruption, but not a loss of the differentiated morphology [51]. In addition, DCs in hypoglycemic medium may be stabilized by a compensatory increase in GLUT3 transporter expression and by the L-glutamine present in DMEM [52,53]. This explanation also aligns with our past data demonstrating that cAMP/RA-predifferentiated cholinergic neurons retained a mature phenotype throughout three consecutive culture passages in media without differentiation factors [11]. Therefore, hypoglycemia induced cell death and decreased viability and cholinergic/acetyl-CoA parameters in NCs and DCs, which may be due exclusively to it limiting the glucose/pyruvate supply, not the absence of differentiation signals (Figure 2, Figure 3 and Figure 4) [11].

The data presented here revealed that at 1–2 mM glucose, DCs display up to 60–100% higher LDH activity compared to NCs (Figure 2G). Under similar conditions, other neuronal cell lines have also demonstrated differentiated characteristics: elevated glucokinase/hexokinase and LDH activities, and increased glycolytic flux (Figure 2G) [34,42,54,55,56]. This explains why, under hypoglycemic conditions, the glycolytic flux in DCs was faster than in NCs (Figure 3A) and thus the glucose AC**_50_** for DCs was lower and the LDH content was higher compared to NCs (Figure 2 and Figure 3). These results are supported by the findings from other studies that showed that higher LDH activity is accompanied by increased glycolytic flux, activating the key steps of this pathway and the TCA cycle due to the increased NAD/NADH**^+^** ratio [46,57]. On the other hand, low or no LDH activity results in slowing of glycolysis due to the decrease in the NAD/NADH**^+^** ratio, resulting in inhibition of glyceraldehyde-3-P dehydrogenase. Therefore, higher LDH activity in hypoglycemic DCs compared to NCs is expected to be accompanied by higher cell counts and a lower proportion of TB+ cells (Table 1, Figure 2). These findings indicate that differentiated cholinergic neuronal septal cells may be better at coping with hypoglycemia compared to those with low expression of the cholinergic phenotype. These findings may also be due to hypoglycemia evoking 70% inhibition of ChAT activity in DCs. This suppression of ACh synthesis might leave more acetyl-CoA for energy production (Figure 3A and Figure 4A) [17,25]. The separate but parallel correlation plots of viability parameters in NCs and DCs indicate the existence of quantitative but not qualitative differences in their reactions to hypoglycemia (Figure 5).

Our data are in agreement with a previous study that detected high-affinity GLUT3 in neurons and neuroblastomas with a Km equal of 1.5–2.5 mM, which was one of the main glucose transporters [49]. This explains the accelerated rates of SN56 cell death at DMEM glucose concentrations below 5 mM (Figure 2 and Figure 3) [58,59,60]. In fact, in SN56 cholinergic neurons that survived 24 h of hypoglycemia, the LDH specific activities were about 80% lower than those cultured in high-glucose media (Figure 2A). This aligns with the hypoglycemia-induced decreases in expression of glycolytic genes in chronically hypoglycemic brains [61]. As a result, metabolic flow through the glycolytic pathway slows down, yielding less pyruvate as a substrate for acetyl-CoA synthesis by neuronal PDHC (Figure 2, Figure 3 and Figure 4) [3,17]. This finding is also in accordance with past studies testing inhibition of glycolysis in brains in both acute and chronic in vivo and in vitro experiments [62,63]. The acetyl-CoA deficit would limit metabolic fluxes through the TCA cycle and respiratory chain, resulting in lower ATP levels. ATP is necessary for the maintenance of neuronal cell growth, axonal transport, and transmitter functions (Figure 2A,B and Figure 4B) [17,64]. These findings are in agreement with those from studies on cultured primary neuronal cells that demonstrated concordant decreases in hexokinase and neuronal enolase activities and overall glycolysis rate under hypoglycemic conditions [65]. However, in this work, predifferentiated DCs displayed higher LDH activity than NCs and a higher overall glycolysis rate compared to previously reported rates (Figure 2G) [56,66]. At 1 mM glucose, there was higher influx of pyruvate into PDHC in DCs than in NCs, and a higher survival rate in DCs (Figure 2E).

The observed higher LDH activity in DCs compared to NCs at low [glucose] levels indicates a higher demand for LDH to produce pyruvate-derived acetyl-CoA, which is necessary for differentiation-activated ACh synthesis/release and maintenance of the membrane potential (Figure 2A,D and Figure 3B) [17,25]. Similar differentiation-related alterations were reported in murine Neuro-2a and human BE(2)-M17 neuroblastomas. Their treatment with RA caused increases in hexokinase and neuron-specific enolase activities and glycolytic gene expression, along with morphological maturation [66,67,68]. In addition, differentiation of several human neuroblastoma cell lines using Nerve Growth Factor (NGF) or 12-O-tetradecanoylphorbol-13-acetate (TPA) increased glycolytic parameters [69,70]. Thus, differentiation-evoked activation of glycolysis might be an adaptative reaction to meet the demands of increased transmitter functions in mature neuronal cells, irrespective of the differentiation signal (Figure 2A) [56,66]. This observation aligns with the responses observed in the non-neuronal HeLa and MCF-7 cell lines, in which hypoglycemia-evoked death was accompanied by increases in not only LDH activity, but also hexokinase, HPI, PFK-1, and GAPDH activities, thereby activating the entire glycolytic pathway [71].

We propose the following mechanisms for the protection of highly differentiated cholinergic neurons during hypoglycemic episodes. Acetyl-CoA is provided from pyruvate produced by LDH from the lactate taken up directly from the extracellular compartment (Figure 2C–F) [39,46]. High LDH activity in hypoglycemic DCs could make them more resistant than NCs to the excitotoxic insults evoked by those conditions (Table 1). This would be particularly important for the cholinergic brain septal neurons responsible for cognitive functions [1,2,3]. This finding is concordant with those of clinical *post-mortem* studies of AD patients that revealed decreased ChAT, ACLY, and PDHC activities in brain areas containing cholinergic neuron terminals that are involved in cognitive functions [14]. Thus, these experiments on SN56 hybrid neuronal cells of septal origin provide a good preliminary basis for studying cholinergic encephalopathy mechanisms in AD or hypoglycemia/hypoxia-evoked cognitive deficits (Table 1, Figure 3 and Figure 4).

Acetyl-CoA is a pivotal molecule in energy metabolism, connecting various potential energy substrates with the TCA cycle/respiratory chain [17,25]. Therefore, maintaining stable levels is a key regulatory target in neuronal cell energy homeostasis. Here, we demonstrate that the 25-fold decrease in [glucose] that resulted in a 60–80% decrease in viability and cytoplasmic enzymatic parameters in cholinergic SN56 neuronal cells only evoked 20 and 30% decreases in PDHC activity and whole-cell [acetyl-CoA], respectively (Figure 1, Figure 2D and Figure 3A). This might be due to the fact that, in cholinergic neurons, the whole-cell acetyl-CoA level is dependent on the rate of its synthesis by mitochondrial PDHC and cytoplasmic ACL/ACS reactions, and its utilization in the TCA cycle, as well as in diverse intra- and extramitochondrial acetylation reactions, including those involved in N-acetyl-aspartate and ACh synthesis, respectively [25]. Hypoglycemic energy deficits may also activate Ca-dependent PTP, resulting in direct efflux of acetyl-CoA to the slow-turnover cytoplasmic compartment [72]. This was evidenced by our results showing that conditions that increase intraneuronal Ca levels markedly decreased intramitochondrial levels of acetyl-CoA and increased cytoplasmic levels of acetyl-CoA but not the whole-cell metabolite pool (Figure 4B) [17,73]. In addition, weaker inhibition of acetyl-CoA synthesis rather than decreased utilization may paradoxically stabilize its whole-cell level (Figure 3A–C and Figure 4A). Another factor supporting this idea of hypoglycemic acetyl-CoA homeostasis is the increased expression of GLUT3 observed in hypoglycemic brains [53,54].

To our knowledge, this is the first report that measured neuronal acetyl-CoA levels under hypoglycemic and neurotoxic conditions (Figure 4 and Figure 6, Table 1). It should be highlighted that effects of SNP and Aβ on SN56 cells in standard DMEM are similar to those reported in other studies [25] for review. This indicates that the results for 1 mM glucose are also reliable (Figure 2, Table 1) and that the acetyl-CoA level is lower in hypoglycemic DCs than in NCs due to higher expression of the cholinergic phenotype [25]. Despite this, DCs showed better survival compared to NCs, presumably due to the supply of pyruvate from exogenous lactate due to having high LDH activity (Figure 2 and Figure 4, Table 1).

The quantal release of ACh by DCs requires instant reconstitution of a releasable pool of ACh and restoration of the membrane potential to maintain cholinergic neurotransmission. In fact, ChAT activity and ACh synthesis in DCs were several times higher than those in NCs (Figure 2D) [11,73]. Therefore, the 40% lower [acetyl-CoA] in DCs could be explained by its additional utilization by ChAT for ACh synthesis, which was several times higher in DCs (Figure 2D and Figure 3A) [17,25,74]. The acetyl-CoA levels in NCs and DCs grown in standard hyperglycemic media were higher than those cultures in hypoglycemic DMEM, which aligns with in vivo studies that demonstrated elevated acetyl-CoA and ACh levels in brain synaptosomes from streptozotocin diabetic rats compared to normoglycemic controls [21]. This indicates that our clonal neuronal cell model adequately reflects the conditions that cholinergic neurons of hyperglycemic or hypoglycemic patients are subjected to [22]. Animal and cell culture studies have found a tight direct correlation between ChAT activity and ACh levels in specific nuclei of the rat brain and cultured neurons [71,72,73,74,75,76,77]. These results are supported by the significant inverse correlation between ChAT activity and [acetyl-CoA] observed in this study (Figure 6E) and earlier findings in different clones of the SN56 cell line [78]. We also found that DCs grown in standard DMEM are more prone to diverse pathogenic signals such as Al or Zn excitotoxicity, excess NO, or thiamine deficiency compared to NCs. All these signals suppress PDHC and acetyl-CoA synthesis (Table 1) [17,25]. This was evidenced here by the higher critical glucose IC**_50_** values for trypan blue retention and LDH release. In addition, the AC_50_ values for ChAT activity were 11.04, 1.85, and 3.85 mM in DCs, and 8.31, 1.01, and 0.68 mM in NCs (Figure 2D,H and Figure 3D).

These results also justify the conclusion that the decrease in ChAT activity in hypoglycemia indicates ACh transmission deficits (Figure 2D). This claim is supported by previous studies that found highly significant correlations between ChAT activity and intracellular and releasable ACh pools [17,74]. Here, we found strong direct correlations between [acetyl-CoA] and [ATP] and intracellular LDH activity, PDHC activity, and death rate in SN56 DCs. This indicates that the glycolysis-dependent supply of pyruvate for PDHC plays a key role in maintaining cholinergic cell viability (Figure 2C and Figure 6D,F). On the other hand, metabolic turnover of cytoplasmic acetyl-CoA is several times slower than in mitochondria, which feeds the TCA cycle to synthesize ATP [25]. This could explain the lack of a significant correlation between ACLY and ChAT activities and cell viability markers within the tested range of glucose concentrations (Figure 6F). However, the significant direct correlation between ChAT activity and acetyl-CoA levels in DCs proves that acetyl-CoA upregulates the expression of ChAT, increasing the energy load and transcription rate (Figure 6E) [25,79]. Differentiation increases acetyl-CoA efflux from mitochondria to the cytoplasmic compartment in SN56 cells [25]. Therefore, it could also stimulate nuclear acetylation, increasing the expression of the *ChAT* gene [80].

The hypoglycemia-evoked decreases in SN56 DC cell counts, ATP levels, and ChAT activity were 3–5 times greater than the decrease in [acetyl-CoA] but they displayed significant mutual correlations (Figure 2F, Figure 3 and Figure 6A–F). In NCs, these correlations were much weaker, likely due to the small changes in the already low basal ChAT activity, resulting in minimal alterations to acetyl-CoA utilization for ACh synthesis (Figure 2C,D and Figure 4A) [9,25]. The nearly proportional alterations and direct, significant correlations between PDHC activity and [acetyl-CoA] indicates that PDHC plays a principal role in stabilizing energy homeostasis in SN56 DCs with high expression of the cholinergic phenotype (Figure 6C).

Hence, the maintenance of a stable level of acetyl-CoA within a wide range of glucose concentrations indicates the existence of strong homeostatic mechanisms. These may support the transmitter functions and survival of septal cholinergic neurons under highly variable extracellular glucose levels [41,42,43]. These mechanisms are critical since numerous enzymes important for neuron survival and function, such as ChAT and aspartate acetyltransferase, have relatively weak affinity for acetyl-CoA, whose intraneuronal concentrations are lower than 10–15 µM [17,25].

Both hypo- and hyperglycemic conditions in vivo may trigger acute secondary pathogenic effects such as glutamatergic excitotoxicity and Ca^2+^ overload. In the long term, this could increase Aβ synthesis and hyperphosphorylation of tau proteins in postsynaptic neurons [59,81,82,83], resulting in over-activation of glutamatergic terminals and an excess of glutamate/zinc in the synaptic cleft. These, in turn, stimulate Ca-dependent NO synthesis, resulting in excessive generation of nitrozyl radicals in postsynaptic neurons [17]. In the present study, the SNP and Aβ treatments were used to generate similar conditions in hypoglycemic SN56 cultures in vitro (Table 1) [11].

These experiments showed that in extremally hypoglycemic conditions, the suppression of ACh synthesis and higher rate of glycolysis in DCs could make them more resistant to cytotoxicity than NCs (Figure 2E,F, Table 1).

**Figure 7:**
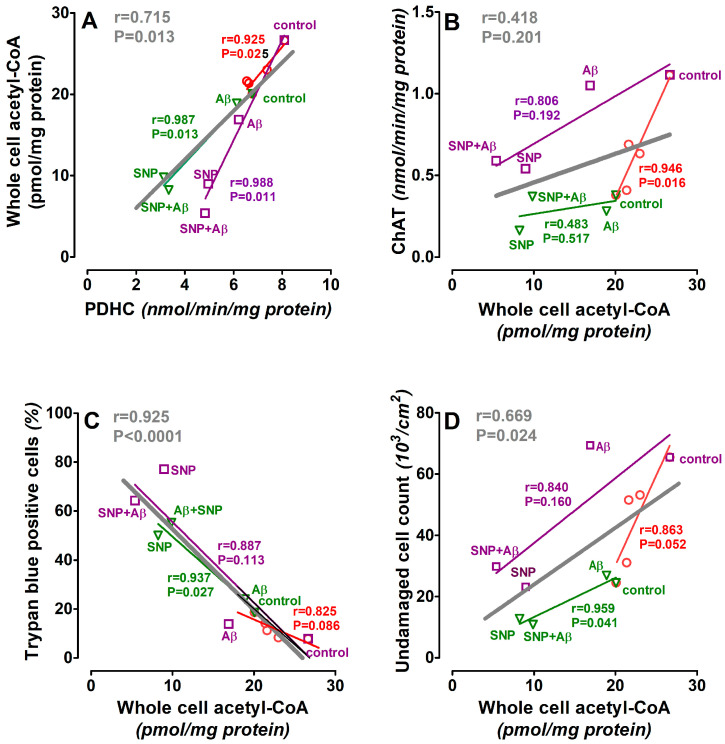
Effects of SNP and Aβ on DCs under hypo- and hyperglycemic conditions. Correlation plots: (**A**) acetyl-CoA vs. PDHC activity; (**B**) ChAT activity vs. acetyl-CoA; (**C**) viability vs. acetyl-CoA; (**D**) cell count vs. acetyl-CoA. Glucose concentrations (1–25 mM) indicated by red lines, SNP and Aβ at 25 mM glucose indicated by purple lines, and SNP and Aβ at 1 mM glucose indicated by green lines. Data taken from Table 1 and Figure 2, Figure 3 and Figure 4.

At the optimal [glucose] (25 mM), both SNP and Aβ, directly or indirectly, inhibited a number of enzymes involved in acetyl-CoA and energy metabolism including PDHC, isocitrate dehydrogenase, and aconitase (Table 1) [25,84]. In addition, they decreased the metabolic and viability parameters of DCs to a greater degree but in a similar manner as hypoglycemia, as demonstrated by the statistically significant correlations between acetyl-CoA level and PDHC or ChAT activity within the tested glucose concentration range (Figure 7). However, at 1 and 25 mM glucose concentrations, the SNP and Aβ effects displayed different slopes, respectively (Figure 7C). This indicates that PDHC inhibition, through limiting the amount of acetyl-CoA provided to mitochondria, indirectly affects ACh synthesis in the cytoplasm of cholinergic neurons (Figure 7A,B) [25].

However, the mechanisms to inhibit the decrease in metabolic flux through PDHC may be different in each case: (i) hypoglycemia decreases the provision of pyruvate through glycolysis inhibition and PDH kinase activation; (ii) SNP/NO removes lipoamide from the E**_2_** subunit; and (iii) Aβ activates PDH kinase and increases Ca influx into mitochondria [17,84,85].

Hypoglycemia disrupts the conversion of glucose to acetyl-CoA in mitochondria in cholinergic neurons, decreasing intramitochondrial acetyl-CoA metabolic flux through the TCA cycle and the generation of ATP (Figure 2, Figure 3 and Figure 4). In the cytoplasm, citrate and ATP deficits reduce the ACLY metabolic rate and suppress its preferential expression in cholinergic neurons (Figure 2C,D) [17,21]. Such conditions restrict ACLY-dependent provision of cytoplasmic acetyl-CoA that is necessary for ACh synthesis and its vesicular accumulation [17,25,78]. A tight interaction between acetyl-CoA metabolism and cholinergic activity is supported here by the observed strong inverse correlations between ChAT activity and acetyl-CoA levels in hypoglycemic DCs (Figure 6E and Figure 7B) [1,2,3]. In contrast, excitotoxins caused much greater suppression of acetyl-CoA levels than hypoglycemia but a much weaker effect on the cholinergic phenotype, irrespective of glycemia level (Figure 3A and Figure 7B). However, the overall functional integrity of cholinergic DCs may depend on acetyl-CoA availability, as demonstrated by the similar plots of TB+ cells vs. acetyl-CoA content for all the neurodegenerative treatments (Figure 7C). The data indicate that LDH/PDHC and acetyl-CoA may be important factors that differentially downregulate the transmitter functions and viability of cholinergic neurons in response to the changes induced by hypoglycemia and excitotoxic signals (Figure 4 and Figure 7). The higher glucose IC**_50_** values for viability and cholinergic activity in DCs highlights the difference between neurons with high and low expression of the cholinergic phenotype. Lower expression of the ACh-transmitter phenotype could explain the relatively higher resistance of fetal and newborn brain cholinergic neurons to hypoxia and other detrimental conditions [85]. On the other hand, the similar plots of SN56 viability vs. acetyl-CoA in all experimental paradigms indicate that acetyl-CoA may be a key pivotal factor for death or survival of septum-derived cholinergic neuronal cells (Figure 7C). Furthermore, the decrease in pyruvate provision by LDH/glycolysis under hypoglycemic conditions could be solely responsible for the functional and structural deficits in cholinergic transmission in affected brains.

The results of this study are in agreement with previous findings in cell cultures as well as surgical, pharmacological, and genetic animal studies modeling human neurodegenerative diseases [6,20,25,42,61]. They indicate that regional and whole-brain disturbances in metabolic pathways and enzymes involved in acetyl-CoA metabolism affect the content and intraneuronal distribution of acetyl-CoA, resulting in impairment of cholinergic neurons and cognitive deficits in the affected animal. Analogous changes in enzymes and metabolic activities were also detected in human encephalopathic brains [14,25]. For obvious reasons, no direct acetyl-CoA data are available for humans. Nevertheless, these findings and those of this study provide insights into the central acetyl-CoA metabolic cycle and indicate that it is a possible target for pharmacological interventions to treat cholinergic encephalopathies.

## Figures and Tables

**Table 1:** Effects of Aβ and SNP on cell count, viability, and acetyl-CoA metabolism parameters in nondifferentiated and differentiated SN56 cholinergic neuronal cells cultivated in DMEM containing 1 or 25 mM glucose.

	(n)	Nondifferentiated SN56		Differentiated SN56	
		1 mM Glucose	25 mM Glucose	1 mM Glucose	25 mM Glucose
A		***Total cell count* (10** **^3^/cm** **^2^)**			
Control Aβ 0.001 SNP 0.4 Aβ 0.001 + SNP 0.4	(5) (3) (3) (3)	24.0 ± 1.1 *** 23.7 ± 2.2 *** 13.2 ± 2.2 ***^‡^ 11.2 ± 1.9 ***^‡‡^	97.0 ± 7.9 92.3 ± 6.4 44.0 ± 4.6 **^‡‡^** 30.8 ± 3.6 **^‡‡‡^**	29.0 ± 0.7 *** 36.4 ± 5.4 *** 15.9 ± 4.8 **^‡^ 15.8 ± 3.5 **^‡‡^	71.0 ± 5.6 85.0 ± 7.9 39.1 ± 5.8 **^‡‡^** 38.8 ± 5.9 ^‡‡^
B		***Trypan blue-positive cells (%)***			
Control Aβ 0.001 SNP 0.4 Aβ 0.001 + SNP 0.4	(5) (3) (3) (3)	14.0 ± 3.3 * 21.9 ± 2.8 ** 97.0 ± 12.5 ***^‡‡‡^ 100.0 ± 3.7 ***^‡‡‡^	3.3 ± 1.6 6.5 ± 1.3 36.9 ± 1.1 ^‡‡‡^ 36.8 ± 0.3 ^‡‡‡^	18.6 ± 2.9 **^†††^** 16.9 ± 3.4 35.3 ± 4.8 ^†††^*** 57.3 ± 1.8 ^†††^*	7.8 ± 1.4 **^†††^** 13.8 ± 3.4 **^†^**^‡^ 77.1 ± 2.3 ^†††‡‡‡^ 64.3 ± 0.5 ^†††‡‡‡^
C		***Viable cell count* (10** **^3^/cm** **^2^)**			
Control Aβ 0.001 SNP 0.4 Aβ 0.001 + SNP 0.4	(5) (3) (3) (3)	20.2 ± 0.9 *** 18.5 ± 1.7 *** 0.4 ± 0.1 ***^‡‡‡^ 0 ***^‡‡‡^	94.7 ± 7.6 86.3 ± 6.0 27.8 ± 2.9 ^‡‡‡^ 19.5 ± 2.3 ^‡‡‡^	23.6 ± 0.6 *** 30.2 ± 0.9 ***^†^ 10.3 ± 3.1 ^‡^ 6.7 ± 1.5 ^‡‡^	65.5 ± 5.2 **^†††^** 73.3 ± 6.8 **^†^** 9.0 ± 1.3 **^†††^**^‡‡‡^ 13.9 ± 2.1 **^†^**^‡‡‡^
D		***PDHC specific activity (nmol/min/mg protein)***			
Control Aβ 0.001 SNP 0.4 Aβ 0.001 + SNP 0.4	(5) (3) (3) (3)	6.8 ± 0.6 * 6.4 ± 0.52 3.4 ± 0.2 ^‡‡‡^ 5.4 ± 0.4 ^‡‡‡^	9.0 ± 0.6 8.1 ± 0.7 6.5 ± 0.2 ^‡‡^ 4.0 ± 0.4 ^‡‡‡^	6.8 ± 0.3 * 5.8 ±0.3 4.4 ± 0.2 ^‡‡^ 4.7 ± 0.5 ^‡‡^	8.1 ± 0.2 7.3 ± 0.7 6.2 ± 0.4 ^‡^ 5.7 ± 0.4 ^‡^
E		***Acetyl-CoA content (pmol/mg protein*** *)*			
Control Aβ 0.001 SNP 0.4 Aβ 0.001 + SNP 0.4	(5) (3) (3) (3)	34.9 ± 3.8 ** 34.5 ± 5.4 22.6 ± 5.5 ^‡^ 30.8 ± 2.5 *	42.9 ± 2.1 27.9 ± 1.7 ^‡^ 17.6 ± 2.7 ^‡‡‡^ 21.2 ± 2.7 ^‡‡^	20.1 ± 1.9 **^†††^ 15.6 ± 2.4 ^††^ 9.8 ± 5.1 ^‡^ 9.0 ± 0.7 ^†††‡‡‡^	26.7 ± 1.6 ^†††^ 20.9 ± 1.7 ^†^ 10.1 ± 0.1 ^†‡‡‡^ 6.2 ± 0.9 ^†††‡‡‡^
F		***ChAT specific activity (nmol/min/mg protein)***			
Control Aβ 0.001 SNP 0.4 Aβ 0.001 + SNP 0.4	(5) (3) (3) (3)	0.08 ± 0.01 0.08 ± 0.01 0.06 ± 0.01 0.05 ± 0.01	0.13 ± 0.01 0.11 ± 0.02 0.08 ± 0.01 0.04 ± 0.02 ^‡^	0.38 ± 0.04 ***^†††^ 0.26 ± 0.01 ***^††^ 0.22 ± 0.02 **^††‡‡^ 0.21 ± 0.03 **^††‡^	1.11 ± 0.04 **^†††^** 0.99 ± 0.10 ^†††^ 0.72 ± 0.11 ^†††‡‡‡^ 0.78 ± 0.11 ^†††‡^

Compared to respective 25 mM glucose condition: * *p* < 0.05, ** *p* < 0.01, *** *p* < 0.001; compared to respective NCs: ^†^ *p* < 0.05, ^††^ *p* < 0.01, ^†††^ *p* < 0.001; compared to respective control: ^‡^ *p* < 0.05, ^‡‡^ *p* < 0.01, ^‡‡‡^ *p* < 0.001. Data are the mean ± SEM from 3–5 independent experiments (given in parentheses).

## Data Availability

Requests for further information and resources should be directed to the lead contact: Sylwia Gul-Hinc (sgul@gumed.edu.pl).

## Abbreviations

Aβ	amyloid-β**_25-35_**
ACh	acetylcholine
ACLY	ATP-citrate lyase
AD	Alzheimer’s disease
ChAT	choline acetyltransferase
dbcAMP	dibutyryl cyclic adenosine 5′ monophosphate
DCs	differentiated SN56 cholinergic neuronal cells
DMEM	Dulbecco’s Modified Eagle Medium
EDTA	ethylenediaminetetraacetic acid
GLUT3	glucose transporter 3
HEPES	2-hydroxyethylpiperazine-‘-2-etanosulfonic acid
[IC_50_]	half-maximum inhibitory concentration
LDH	lactate dehydrogenase
NCs	nondifferentiated SN56 cholinergic neuroblastoma cells
PDHC	pyruvate dehydrogenase complex
RA	retinoic acid
SNP	sodium nitroprusside
TB+	trypan blue retention assay
